# Hsp70-Hsp90 organising protein (HOP/STIP1) is required for KSHV lytic replication

**DOI:** 10.1099/jgv.0.002053

**Published:** 2024-11-28

**Authors:** Elisa Kirigin, Michael Obinna Okpara, Lorraine Matandirotya, Jamie-Lee Ruck, Frederick Weaver, Zoe Jackson, Abir Chakraborty, Clinton Gareth Lancaster Veale, Adrian Whitehouse, Adrienne Lesley Edkins

**Affiliations:** 1 Biomedical Biotechnology Research Unit (BioBRU), Department of Biochemistry and Microbiology, Rhodes University, Makhanda, 6139, South Africa; 2 School of Molecular and Cellular Biology, Faculty of Biological Sciences, University of Leeds, Leeds, LS2 9JT, UK; 3 Department of Chemistry, University of Cape Town, Rondebosch, Cape Town, 7700, South Africa; 4 Centre for Chemico- and Biomedicinal Research (CCBR), Rhodes University, Makhanda, 6139, South Africa

**Keywords:** chaperone, HOP/STIP1, KSHV, viral lytic replication

## Abstract

Kaposi’s sarcoma-associated herpesvirus (KSHV) is a DNA virus that causes Kaposi’s sarcoma, a cancer of endothelial origin. KSHV uses the activity of host molecular chaperones like Hsp70 and Hsp90 for the folding of host and viral proteins required for productive infection. Hsp70 and Hsp90 chaperones form proteostasis networks with several regulatory proteins known as co-chaperones. Of these, Hsp90–Hsp70-organizing protein (HOP) is an early-stage co-chaperone that regulates the transfer of folding substrate proteins between the Hsp70 and Hsp90 chaperone systems. While the roles for Hsp90 and Hsp70 in KSHV biology have been described, HOP has not previously been studied in this context despite its prominent interaction with both chaperones. Here, we demonstrate a novel function for HOP as a new host factor required for effective lytic replication of KSHV in primary effusion cell lines.

## Data Summary

The authors confirm that all supporting data, code and protocols have been provided within the article or through supplementary data files.

## Introduction

Kaposi’s sarcoma-associated herpesvirus (KSHV; also known as human herpesvirus 8) is a DNA oncovirus required for the development of the endothelial tumour Kaposi’s sarcoma (KS) and the lymphoproliferative disorders primary effusion lymphoma and multicentric Castleman’s disease [1]. KSHV, like all herpesviruses, establishes a persistent life-long infection in the host known as latency, during which the viral episome is maintained through tethering to the host genome. During latency, only a few viral genes, including the latency-associated nuclear antigen (LANA) are expressed. KSHV undergoes periodic episodes of lytic reactivation characterized by the expression of numerous viral transcripts, increased DNA replication and assembly and budding of infectious virions. The first transcript to be expressed is ORF50, which encodes the replication and transactivator (RTA) protein that initiates the lytic cycle [2,3]. Early genes, which are expressed 10–24 h post-induction and encode functions that mediate viral gene expression and DNA replication, include ORF47 and ORF57. At 24 h post-reactivation, KSHV expresses late genes, which primarily encode structural components such as capsid (e.g. ORF65) and tegument proteins [2,4, 5]. Both the latent and lytic life cycles of KSHV contribute to oncogenesis [5]. The inhibition of lytic replication is considered one potential therapeutic strategy, mainly by targeting the viral DNA polymerase [6,7]. However, host factors that regulate the lytic cycle may also represent potential targets for therapeutic interventions. For example, previous reports have linked cellular kinases including mTOR to productive KSHV lytic replication and shown that the inhibition of the associated host pathways with rapamycin reduces lytic replication [8].

KSHV lytic replication is characterized by rapid sequential production of multiple viral transcripts, most of which must be translated into protein. Many viral proteins require the chaperone protein folding systems of the host cell to reach a functional state and hence can be characterized as ‘clients’ [9]. The largest group of molecular chaperones are members of the heat shock protein (HSP) family and function in an ATP-dependent manner to support folding, prevent aggregation and regulate degradation of client proteins [9]. Viral proteins are often multifunctional and more structurally complex than cellular proteins, and the rapid synthesis of high concentrations of multiple proteins simultaneously by viruses leaves proteins vulnerable to aggregation and misfolding and increases reliance on molecular chaperones [10,11]. Consequently, many viral proteins are exquisitely sensitive to perturbation of host chaperone systems even despite high similarity to human homologues [12], and consequently, the inhibition of viral protein homeostasis can be achieved while retaining sufficient proteostasis capability to support cell viability.

Molecular chaperones like Hsp90 and Hsp70 regulate KSHV biology by controlling the stability of key KSHV client proteins [4,13,15]. Hsp90 interacts with and stabilizes a wide range of client proteins, many of which are linked to oncogenesis and show diversity in amino acid sequences and functions, including kinases, E3 ligases and transcription factors [16]. While Hsp90 is mainly involved in conformational stabilization of unstable conformations and binds folded proteins, folding intermediates and disordered proteins [17], Hsp70 isoforms primarily catalyse *de novo* and stress-related folding of numerous cellular proteins [18]. Hsp90 and Hsp70 are associated with KSHV virions [19,20], and Hsp90 interacts with important KSHV proteins, including LANA, K1 and vFLIP, which are degraded in response to Hsp90 inhibition [13,15].

In addition, the inhibition of chaperones can also degrade host cell proteins essential for viral infection, including the KSHV entry receptor Ephrin A2 [13]. Hsp70 isoforms (both cytoplasmic and organelle associated) are linked to productive viral replication in multiple viruses, including KSHV [4,21, 22]. Hsp70 isoforms are recruited into nuclear KSHV viral replication and transcription centres (RTCs), and Hsp70 ATPase activity is required to support viral DNA replication and gene expression and ORF57 function [4]. While chaperones are involved in maintaining latency, KSHV lytic replication substantially increases the protein folding burden in the host cell, creating a demand for chaperone folding. Consequently, many viral proteins are highly susceptible to aggregation when host cell folding pathways are blocked. Therefore, selective inhibition of chaperone activity can have potent antiviral effects [10,23, 24].

Hsp90 and Hsp70 systems collaborate during protein folding, creating a higher-order folding network that includes regulatory cofactors known as co-chaperones. The Hsp90–Hsp70-organizing protein (HOP; also known as p60, sti1 or STIP1) is a co-chaperone that coordinates the exchange of client proteins between the Hsp90 and Hsp70 chaperone systems [25,26]. While HOP is not required for client transfer between Hsp70 and Hsp90, it alters the rate of client exchange and chaperone-mediated folding [27]. HOP is upregulated in many cancerous states where it may also be constitutively associated with Hsp90 and Hsp70 complexes [28]. The depletion of HOP levels is effective at reversing the cancer phenotype, although these studies have been conducted in adherent cell types [29,33]. Both Hsp90 and Hsp70 are important to virus function, and HOP plays a regulatory role in assembling Hsp70–Hsp90 chaperone-client complexes. HSP90–KSHV-containing complexes isolated from KSHV-infected B cells also contain HOP [14]. We therefore hypothesized that HOP may also be important in KSHV biology. If this could be established, then HOP may represent a new host target for anti-KSHV therapies and a potential strategy to simultaneously perturb both Hsp70 and Hsp90 functions.

## Methods

### Maintenance of cell lines

The BJAB (ACC 757) (EBV^−^/KSHV^−^), BCBL-1 (CVCL_0165) (EBV^−^/KSHV^+^), BC-1 (CRL-2230) (EBV^+^/KSHV^+^) and Raji (CCL-86) (EBV^+^/KSHV^−^) lymphoma cell lines were maintained in Roswell Park Memorial Institute (RPMI) 1640 medium (Thermo Fisher, 21875091), supplemented with 10% (v/v) FBS (FBS-GI-HI-12A, Biocom Africa), for BJAB, BCBL-1 and Raji or 20% FBS for BC-1, 1% (v/v) penicillin/streptomycin (17-745E, Lonza) and 1% (v/v) GlutaMAX at 37 °C with 5% CO_2_. The TREx BCBL-1-RTA cells (developed by Dr Jae Jung, University of Southern California) are the BCBL-1 cell line containing a doxycycline-inducible RTA transcriptional activator, which acts as the molecular switch for KSHV lytic reactivation [34]. The TREx BCBL1-RTA cells were maintained in RPMI 1640 with 10% (v/v) FBS, 1% (v/v) penicillin/streptomycin, 1% (v/v) GlutaMAX, 100 µg ml^−1^ hygromycin B (GA7834, Glentham Life Sciences) at 37 °C with 5% CO_2_. To generate stable cell lines expressing shRNA for HOP (or shNT control) or overexpressing GFP and HOP–GFP, the TREx BCBL1-RTA cells were transduced with the shRNA or overexpression plasmids (supplementary data) [35]. The cells were maintained in the TREx BCBL1-RTA medium plus 3 µg ml^−1^ puromycin (P7255, Sigma-Aldrich) at 37 °C with 5% CO_2_. The HEK293T cell line was maintained in Dulbecco’s Modified Eagle’s Medium (DMEM) with 10% (v/v) FBS, 1% (v/v) GlutaMAX, 1% (v/v) penicillin/streptomycin, 1% (w/v) G418 (Glentham Life Sciences, GA2946), 1% (v/v) non-essential aas (Lonza, BE13-114E) and 1% (v/v) sodium pyruvate (Sigma-Aldrich, S8636) at 37 °C in 9% CO_2_. The HEK293T rKSHV.219 cell line contains KSHV.219, a recombinant form of the KSHV genome and a gift from Dr Jeffery Vieira (University of Washington Seattle) [36]. The cells were grown in DMEM with 10% (v/v) FBS, 1% (v/v) penicillin/streptomycin (100 µg ml^−1^) and puromycin (2 µg ml^−1^) for the selection of the KSHV genome.

### Induction of KSHV lytic replication

To induce KSHV lytic replication in the BCBL-1 cell line, the cells were synchronized at the G1/S phase by a double thymidine block [37]. The cells were seeded at 40% confluency in a medium containing 2 mM thymidine for 18 h, before being washed with fresh medium, cultured for 9 h and treated with 2 mM thymidine for 18 h. After the second thymidine treatment, the cells were washed with fresh medium, and lytic reactivation was induced with 1.5 mM sodium butyrate. The TREx BCBL1-RTA cells were treated with 2 µg ml^−1^ doxycycline hyclate to induce lytic reactivation for the specified times. The HEK293T rKSHV.219 cells or uninfected HEK293T cells were treated to induce lytic reactivation cells with sodium butyrate (4 mM) and tetradecanoylphorbol acetate (TPA) (20 ng ml^−1^). To harvest, the cells were collected by centrifugation at 800 ***g*** for 5 min at 4 °C, washed with sterile PBS and centrifuged at 800 ***g*** for 5 min at 4 °C, and the cell pellet was either used immediately or stored at −20 °C.

### Isolation of PBMCs from whole blood

Ethics clearance (project ID: 1347; review reference: 2020-1347-3509) was granted for the harvesting of PBMCs. BD Vacutainer® CPT™ Mononuclear Cell Preparation Tubes with sodium citrate (BD Biosciences, 362761) were used to isolate PBMCs from whole blood from participants who had given informed consent prior to blood collection. From each participant, 3×8 ml tubes of whole blood were collected and processed as per the manufacturer’s instructions. The samples were centrifuged for 30 min at 1800 xg at room temperature. For each participant, PBMC layers were pooled and lysed in SDS sample buffer [0.05 M Tris-HCl, pH 6.8, 10% (v/v) glycerol, 2% (w/v) SDS and 5% (v/v) *β*-mercaptoethanol] for subsequent Western blot analysis.

### Quantitative polymerase chain reaction (qPCR) and gene expression analysis

RNA was extracted using TRI Reagent and the Direct-zol RNA Miniprep Kit (Zymo Research) and concentrated used the RNA Clean and Concentrator-5 Kit (Zymo Research, catalogue number: R1013). cDNA was synthesized using LunaScript RT Supermix Kit (New England Biolabs), and Luna Universal qPCR Master Mix Protocol was used for qPCR amplification (M3003, New England Biolabs) on the CFX Real-Time PCR detection system (Bio-Rad, USA). The optimized standard thermocycling procedure for qPCR was 95 °C for 1 min, 95 °C for 15 s and 60°C for 30 s for 39 cycles, with a melt curve to confirm specific amplification of a single target sequence. The expression levels of target genes were calculated as relative normalized expression (2^–∆∆Ct^) fold change relative to GAPDH [38]. Details of qPCR primers are in Table S1, available in the online version of this article.

### Viral DNA load quantitation assay

Total DNA was extracted from cell pellets using the Zymo Quick gDNA MiniPrep Kit (D3024). Viral DNA was quantified by qPCR analysis of viral genomic ORF57 amplification relative to human genomic DNA (quantified by amplification of GAPDH) using the 2^–∆∆Ct^ method [38].

### Supernatant transfer assay

A total of 3×10^6^ TREx-BCBL1-RTA cells were seeded in 3 ml of antibiotic-free growth medium supplemented with or without 4 µg ml^−1^ doxycycline for 72 h. Thereafter, the supernatant from TREx-BCBL1-RTA cells was centrifuged at 500 ***g***, filtered through a 0.45-µm filter, mixed with 300 µl of complete HEK293T growth medium and added to adherent HEK293T cells for 24 h. Subsequently, either total DNA or RNA was extracted from the HEK293T pellets and qPCR analysis (as previously described) used to determine the level of ORF57 DNA or mRNA transcript relative to human genomic DNA (quantified by amplification of GAPDH locus) or the GAPDH host reference transcript using the 2^–∆∆Ct^ method [38].

### Western blot analysis

The cells were lysed in Radioimmunoprecipitation lysis buffer (R0278, Sigma-Aldrich) with 1 % (v/v) protease inhibitor cocktail for 45 min on ice. The samples were prepared for SDS-PAGE to achieve equivalent protein concentration by boiling in 1× loading dye [0.05 M Tris-HCl, pH 6.8, 10% (v/v) glycerol, 2% (w/v) SDS, 0.02% (w/v) bromophenol blue and 5% (v/v) *β*-mercaptoethanol] [39]. Proteins were transferred to nitrocellulose membranes using the Turbo-Blot transfer system (Bio-Rad). Membranes were blocked in 1% (w/v) BLOTTO (Santa Cruz Biotechnology) in tris buffered saline with Tween 20 (TBST) [50 mM Tris, 0.15 M NaCl, pH 7.5 and 0.1% (v/v) Tween 20] for 1 h at room temperature and incubated with primary antibody at 4 °C in 1% (w/v) BLOTTO/TBST overnight. Membranes were washed five times for 5 min with TBST and incubated with appropriate HRP-conjugated secondary antibody in 1% (w/v) BLOTTO/TBST for 1 h at room temperature. Membranes were washed as described and protein bands visualized using the enhanced chemiluminescent substrate with the ChemiDoc™ XRS system (Bio-Rad). Details of primary and secondary antibodies are in Table S2.

### Indirect immunofluorescence assay

The BCBL-1 cells remained untreated, or KSHV lytic reactivation was induced with 1.5 mM sodium butyrate for the specified times. The cells were allowed to adhere to poly-l-lysine-coated glass coverslips for 30 min at 37 °C. Coverslips were washed once with PBS and cells fixed with 4% (w/v) paraformaldehyde in PBS for 10 min at room temperature. Coverslips were washed a further three times with PBS, and the cells were permeabilized with 0.1% (v/v) Triton X-100 for 10 min, washed three times with PBS and blocked with 1% (w/v) BSA in PBS for 1 h at 37 °C. Appropriate dilutions of primary antibodies were prepared in 0.1% (w/v) BSA in PBS and incubated with coverslips at 4 °C overnight (Table S2). Coverslips were washed three times with PBS and incubated with fluorescently conjugated species-specific secondary antibodies prepared in 0.1% (w/v) BSA in PBS for 1 h at room temperature (Table S2). Coverslips were washed a further three times with PBS before a final 1-min incubation with nuclear stain (Hoechst 33342 at 1 µg ml^−1^ in distilled water). Coverslips were mounted onto slides with DAKO fluorescent mounting medium (Agilent, S3023) and cells visualized using a Zeiss LSM 780 confocal microscope.

### Cell counting assays to measure cell proliferation

The cells were seeded at a density of 0.4×10^6^ cells per millilitre in 3-ml volumes in six-well culture plates and allowed to grow for 96 h at 37 °C. For the reactivation of KSHV lytic replication in TREx-BCBL-1 cells, the cells were treated with 2 µg ml^−1^ doxycycline at seeding. Growth (cell density) was monitored every 24 h up to 96 h by counting the live cells at 24, 48, 72 and 96 h using a haemocytometer. Trypan blue solution (Sigma-Aldrich) was used to distinguish between the dead and live cells, and only live cells were counted. Cell counts were normalized to the density during seeding (0 h), which was taken as 1.

## Results and discussion

**Fig. 1.**
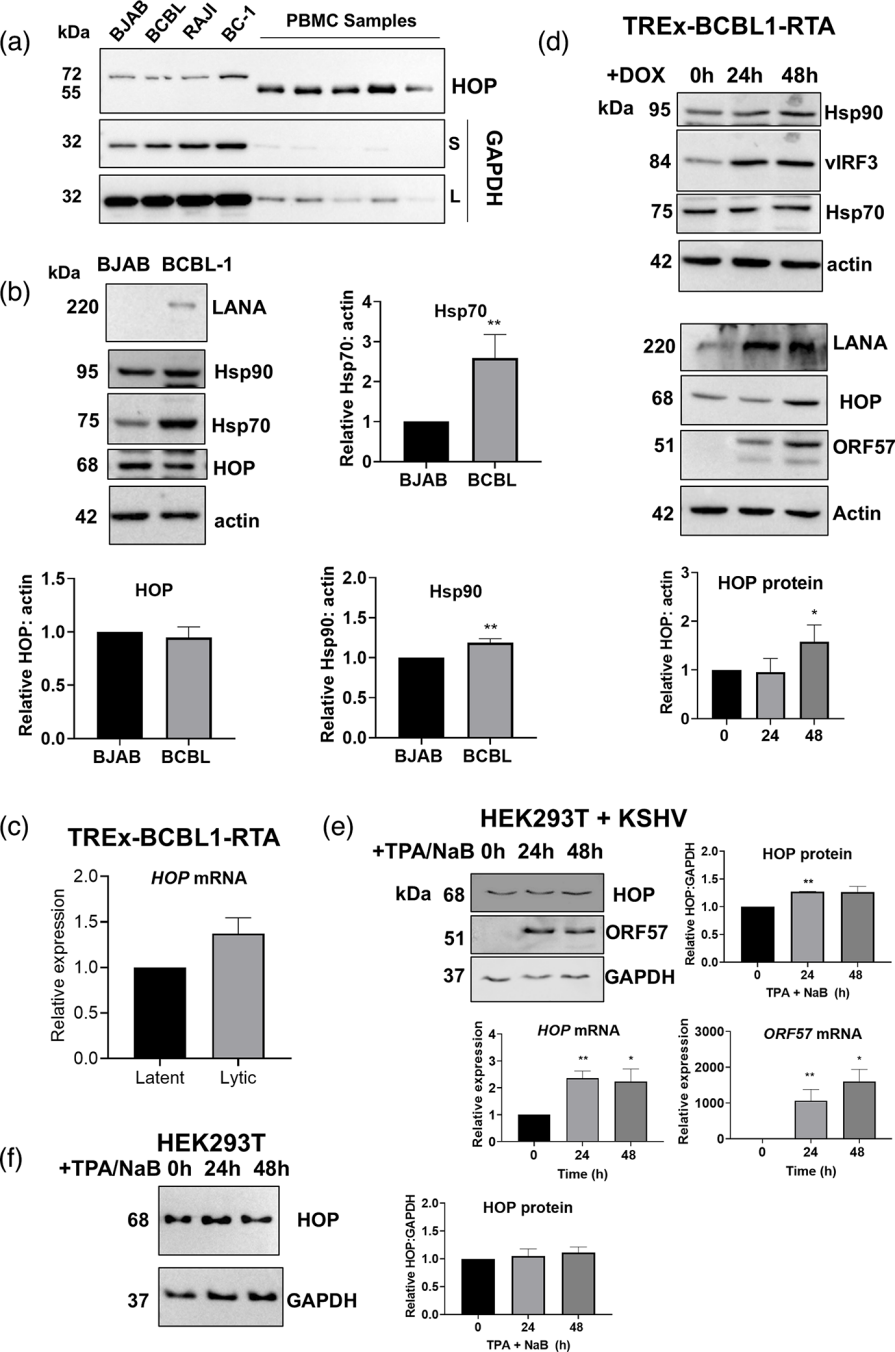
KSHV lytic replication increases HOP levels. (**a)** Comparison of HOP levels in lymphoma cell lines and PBMC by Western blot. BJAB (KSHV^−^/EBV^−^), BCBL (KSHV^+^/EBV^−^), Raji (KSHV^−^/EBV^+^) and BC-1 (KSHV^+^/EBV^+^). GAPDH was used as a loading control. S, short exposure; L, long exposure. (b) Western blot and average densitometry (±sd, *n*=3) analysis of HOP, Hsp70 and Hsp90 levels in uninfected BJAB and KSHV-infected BCBL-1 lymphoma lines. (c) Relative mRNA expression levels of HOP in the TREx-BCBL1-RTA cell line during latency and 24 h after reactivation of lytic replication with doxycycline. (d) Western blot and average densitometry (±sd, *n*=3) analysis of HOP protein levels in the TREx-BCBL1-RTA cell line during latency and after lytic reactivation with doxycycline. (e) Western blot and average densitometry (±sd, *n*=3) analysis of HOP levels in the HEK293T rKSHV.219 cells and relative mRNA expression levels of HOP and ORF57 during latency and 24/48 h after reactivation of lytic replication with sodium butyrate and TPA. (f) Western blot and average densitometry (±sd, *n*=3) analysis of HOP levels in uninfected HEK293T cells treated with sodium butyrate and TPA for 24/48 h. Unpaired t-tests were used to determine statistical significance relative to BJAB or latent samples, where * and ** indicate *P* values of <0.05 and <0.01, respectively.

HOP has not previously been studied in the context of KSHV infection. However, HOP levels were increased in simian virus 40-infected fibroblasts [40,41] and hepatocellular carcinoma cells infected with hepatitis B virus [42]. We first compared the expression levels of HOP in healthy PBMCs and lymphoma lines with and without KSHV infection (BJAB KSHV^−^, BCBL-1 KSHV^+^EBV^−^, BC-1 KSHV^+^EBV^+^ and Raji KSHV^−^EBV^+^) (Fig. 1a). HOP resolved at a lower molecular weight in PBMC samples isolated from normal individuals compared to the lymphoma cell lines. HOP is subject to post-translational modifications, including phosphorylation and acetylation [26,43,45], and this likely explains the size difference of HOP in the cancer cell lines (irrespective of KSHV infection) (Fig. 1a). HOP protein levels were not significantly altered with KSHV infection (comparing the KSHV^−^ BJAB cell line to the BCBL-1 and BC-1 cell lines that have persistent KSHV infection). In contrast, Hsp70 and Hsp90 were significantly upregulated in the BCBL-1 cell line (KSHV^+^EBV^−^) compared to the BJAB (KSHV^−^EBV^−^) line (Fig. 1a, b). This suggested that KSHV infection did not increase basal HOP protein levels, but rather increased Hsp70 and Hsp90 expressions. The increased levels of chaperones may serve to support viral protein homeostasis [46]. Hsp70 and Hsp90 chaperones regulate multiple viral processes, including viral entry, replication, gene expression and viral protein localization [47,51], and hence, increased expression of these proteins with viral infection is not unexpected. The reactivation of KSHV lytic replication by doxycycline-induced expression of RTA for 24 h in the BCBL-1-based TREx-BCBL1-RTA cell line did not significantly increase HOP mRNA expression relative to the GAPDH reference transcript (Fig. 1c). HOP protein levels were significantly increased with lytic induction compared to latency at 48 h post-infection (Fig. 1d). Lytic reactivation via doxycycline had no effect on the *β*-actin loading control. Although Hsp70 protein levels remained constant with lytic induction, Hsp90 protein levels were upregulated at 48 h post-lytic induction. The induction of lytic replication over 48 h also increased the expression of latent proteins LANA and vIRF3, as well as the lytic protein marker ORF57. A minor but significant increase in HOP protein levels was observed after 48 h post-lytic reactivation in the HEK293T rKSHV.219 cell line model, while the increase in HOP mRNA levels was significant from 24 h post-lytic reactivation (Fig. 1e). As expected for a lytic gene, ORF57 mRNA and protein levels in the HEK293T rKSHV.219 cell line were significantly increased relative to latency (Fig. 1e, upper and middle panels). The treatment of uninfected HEK293T cells with sodium butyrate and TPA for 24 and 48 h did not increase the levels of HOP protein, showing that this effect was specific to KSHV-infected cells. This suggested a possible involvement of HOP during KSHV lytic replication, particularly in lymphoma cell lines.

**Fig. 2.**
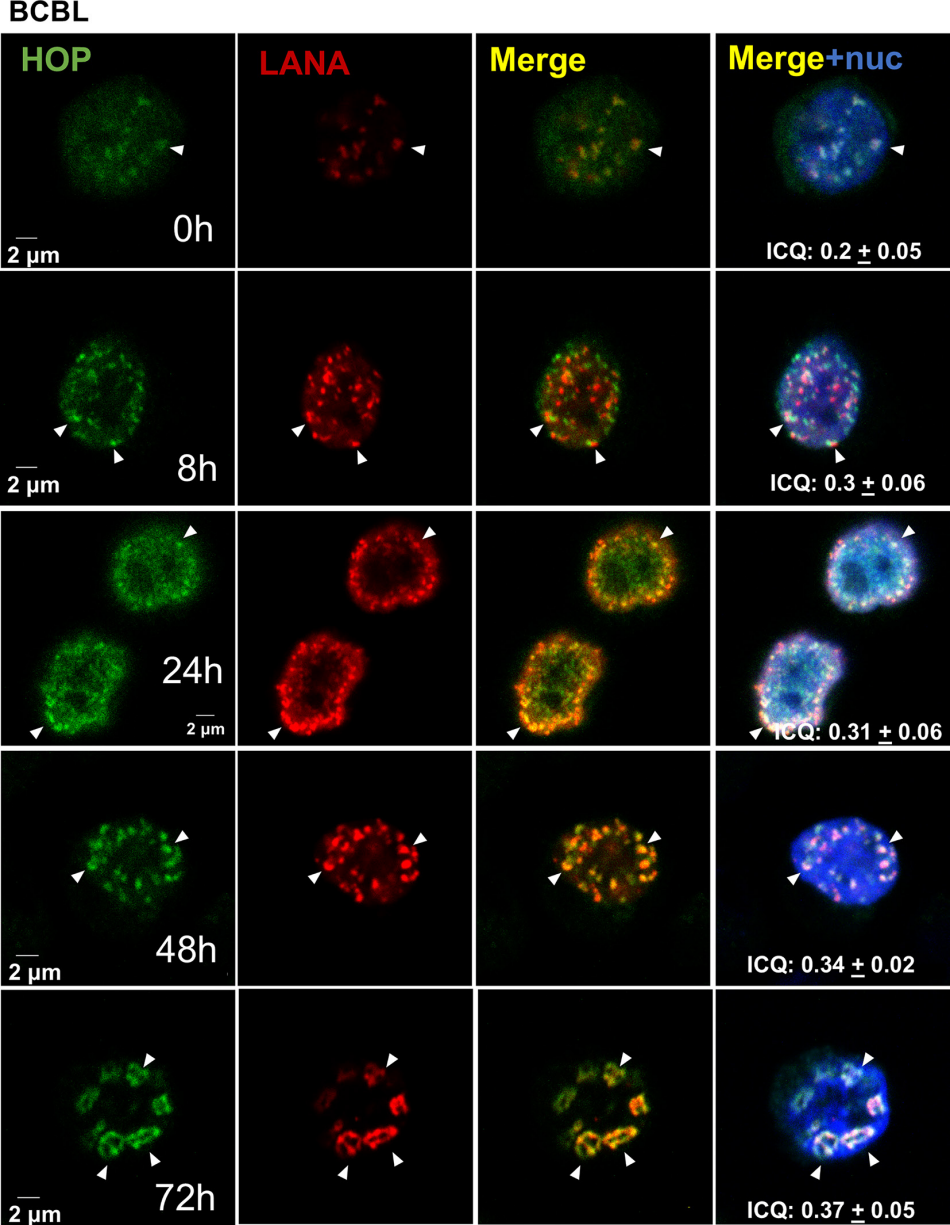
HOP is recruited to KSHV replication compartments. Subcellular localization of HOP (green) and LANA (red) in the BCBL-1 cell line during KSHV lytic reactivation determined using immunofluorescence microscopy. White arrows indicate regions of co-localization of HOP and LANA puncta. Co-localization is shown by the average intensity correlation quotient (ICQ) calculated in ImageJ where a value closer to 0.5 indicates perfect co-localization and a value of −0.5 indicates perfect exclusion. The sd shown is from at least 15 cells analysed over a minimum of 3 independent fields. A double thymidine block was used to synchronize cells to increase the efficacy of viral replication [37]. The cells were harvested for analysis after lytic reactivation for the time points indicated.

KSHV replication compartments form in the nucleus during lytic replication, and Hsp70 is known to be recruited into these structures [4]. Therefore, we next evaluated whether the subcellular distribution of HOP was also altered during KSHV lytic replication. HOP localizes predominantly to the cytoplasm but contains a bipartite nuclear localization signal that permits cell cycle and stress-induced nuclear accumulation regulated by phosphorylation [40,52, 53]. HOP also localizes to the Golgi apparatus, vesicles and extracellular environment depending on cellular context [40,54]. In the BCBL-1 cell line cultured under latent conditions, HOP was predominantly observed in nuclear puncta that localized adjacent to the KSHV episome-associated protein LANA, although some diffuse staining in the cytoplasm was noticeable (Fig. 2). With lytic reactivation, HOP distribution was adjacent to LANA at 8 h and showed a time-dependent increase in nuclear accumulation (and a concomitant loss in the cytoplasmic signal) and increasingly localized with LANA and the viral episome from 24 h post-lytic reactivation. LANA is necessary for both KSHV genome maintenance and high levels of lytic gene expression [55]. By 72 h post-reactivation of lytic replication, HOP showed strong localization with LANA in ring-like KSHV LANA-associated nuclear bodies (LANA-NBs) [56] (Fig. 2). These observations are supported by increased abundance of HOP in isolated KSHV RTC fractions by MS [4]. Taken together, these data suggest that HOP is recruited into LANA-NBs during KSHV replication and hence may participate as a host factor for the viral lytic cycle. To date, there are no reports of HOP interaction with cytoplasmic mammalian viruses; however, previous reports showed that GFP–HOP was recruited into potato virus Y replication complexes in the endoplasmic reticulum (ER) in plants [57]. HOP was also identified in complex with the P2B subunit of influenza A RNA polymerase, but its role in the replication was not studied [58]. Although both examples occur in ssRNA viruses whose life cycles vary greatly from KSHV, this suggests a link between HOP and viral replication.

**Fig. 3.**
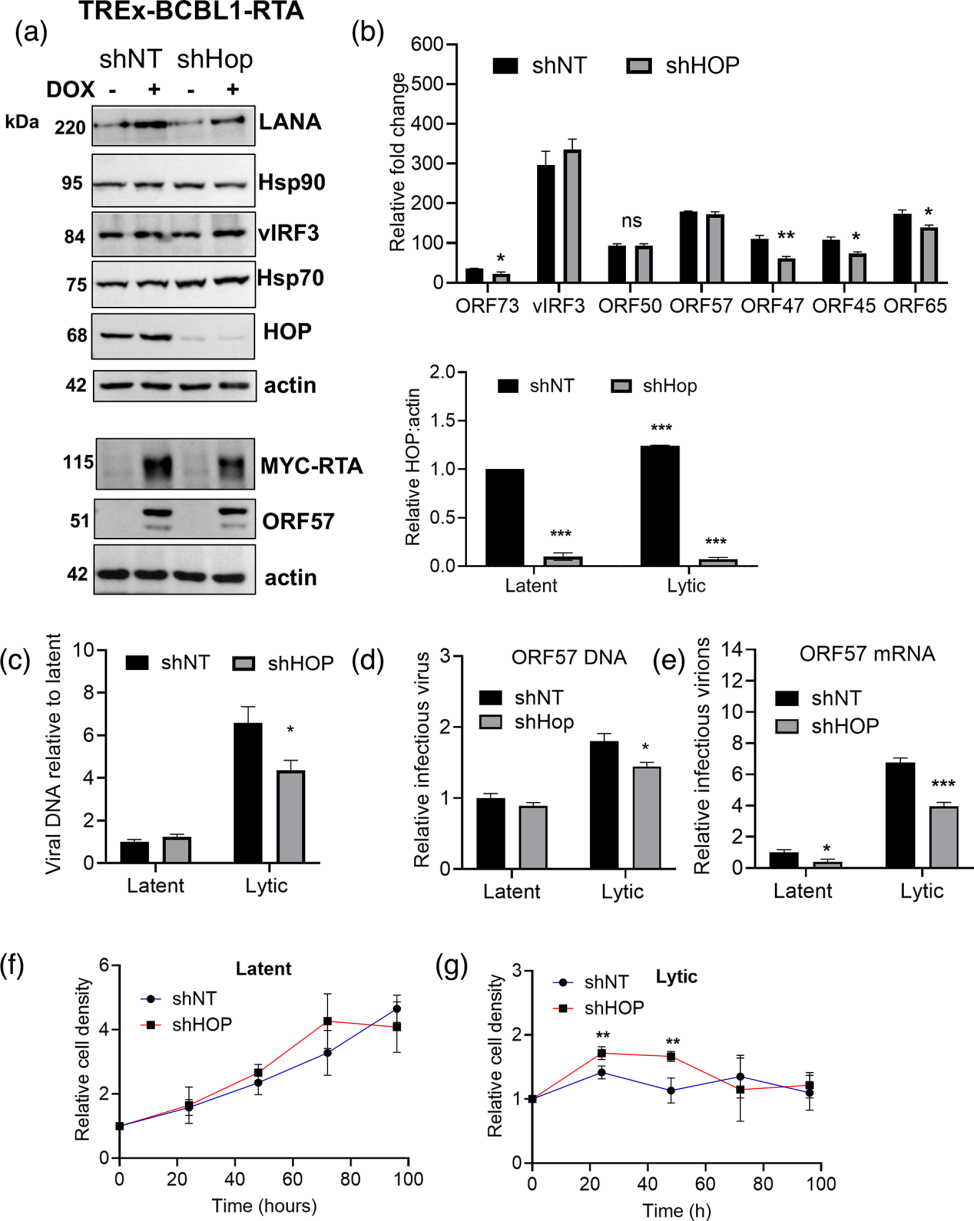
HOP depletion reduced KSHV lytic replication. (**a)** TREx-BCBL-1-RTA cell lines expressing non-targeting control (shNT) or HOP-specific (shHOP) shRNA were treated with doxycycline (dox) to induce lytic reactivation for 24 h before protein levels were confirmed by Western blot analysis. (b) Relative expression of viral genes in shNT and shHOP cell lines at 24-h lytic reactivation. mRNA transcripts were quantified by qPCR analysis of normalized expression relative to the GAPDH reference gene. (c) Quantitation of viral load. TREx-BCBL-1-RTA cell lines were treated with dox to induce lytic reactivation for 72 h or left untreated (latent) and total DNA was isolated. Viral load was assessed by qPCR analysis of viral genomic ORF57 amplification relative to human genomic DNA (quantified by amplification of GAPDH). (d) Viral supernatant transfer assay to quantify infectious virion production. TREx-BCBL-1-RTA cells were treated with dox (lytic) for 72 h to reactivate KSHV lytic replication or left untreated (latent). HEK293T cells were incubated with the culture medium from the untreated and dox-treated cells. Total (e) DNA or (f) RNA was extracted from the HEK293T cells at 24 h post-incubation, and infectious virion production was measured by qPCR analysis of normalized expression of ORF57 relative to the GAPDH reference gene. (d) Data are averages (±sem, *n*=3). Unpaired t-tests were used to determine the statistical significance relative to shNT samples, where *, ** and *** indicate *P* values of <0.05, <0.01 and <0.001, respectively.

To test whether HOP was required for the KSHV lytic cycle, we developed the TREx-BCBL1-RTA cell lines stably expressing either control (shNT) or HOP-specific (shHOP) shRNA to reduce HOP protein levels. Robust depletion of HOP was achieved in the shHOP cell line (Fig. 3a) The cell lines were cultured under latent conditions or lytic reactivation induced by the production of RTA in response to the addition of doxycycline. Protein levels of Hsp90 were unchanged with HOP depletion, while protein levels of Hsp70 were upregulated with HOP depletion, suggesting an upregulation of the stress response. During lytic replication conditions, LANA protein levels were reduced with HOP depletion, whereas vIRF3 and ORF57 levels were unchanged with HOP depletion (Fig. 3a). To monitor the KSHV lytic cycle, we assessed the levels of viral mRNA transcripts relative to the host housekeeping gene GAPDH by qPCR (Fig. 3b), viral DNA load relative to genomic DNA by qPCR (Fig. 3c) and production of infectious virions by monitoring reinfection of naïve HEK293T endothelial cells (Fig. 3d). HOP depletion reduced the expression of multiple KSHV lytic transcripts (including ORF47, ORF45 and ORF65) in addition to reducing the expression of the latent gene ORF73 (LANA). This was not due to the differences in the induction of lytic reactivation since there was no significant difference in the levels of ORF50/RTA transcripts between the shNT and shHOP lines. Although the ORF50 primers used did not differentiate between the endogenous RTA encoded in the KSHV genome and the tetracycline-inducible RTA transgene, protein levels of inducible MYC–RTA were consistent in both the control and shHOP cell lines (Fig. 3a), confirming that the lytic induction was equivalent. In addition, HOP depletion significantly reduced viral DNA in the TREx-BCBL1-RTA cell line (Fig. 3c) and significantly reduced the production of infectious KSHV virions during lytic replication (Fig. 3d, e). HOP depletion had no significant effect on cell growth during latent conditions compared to the shNT control (Fig. 3e) but marginally increased cell growth during lytic replication at early time points (24 and 48 h post-seeding) (Fig. 3f). This suggested that the presence of HOP could support viral lytic replication and consequently inhibit host cell growth, while HOP depletion could reduce viral progression to allow higher host cell proliferation rates.

**Fig. 4.**
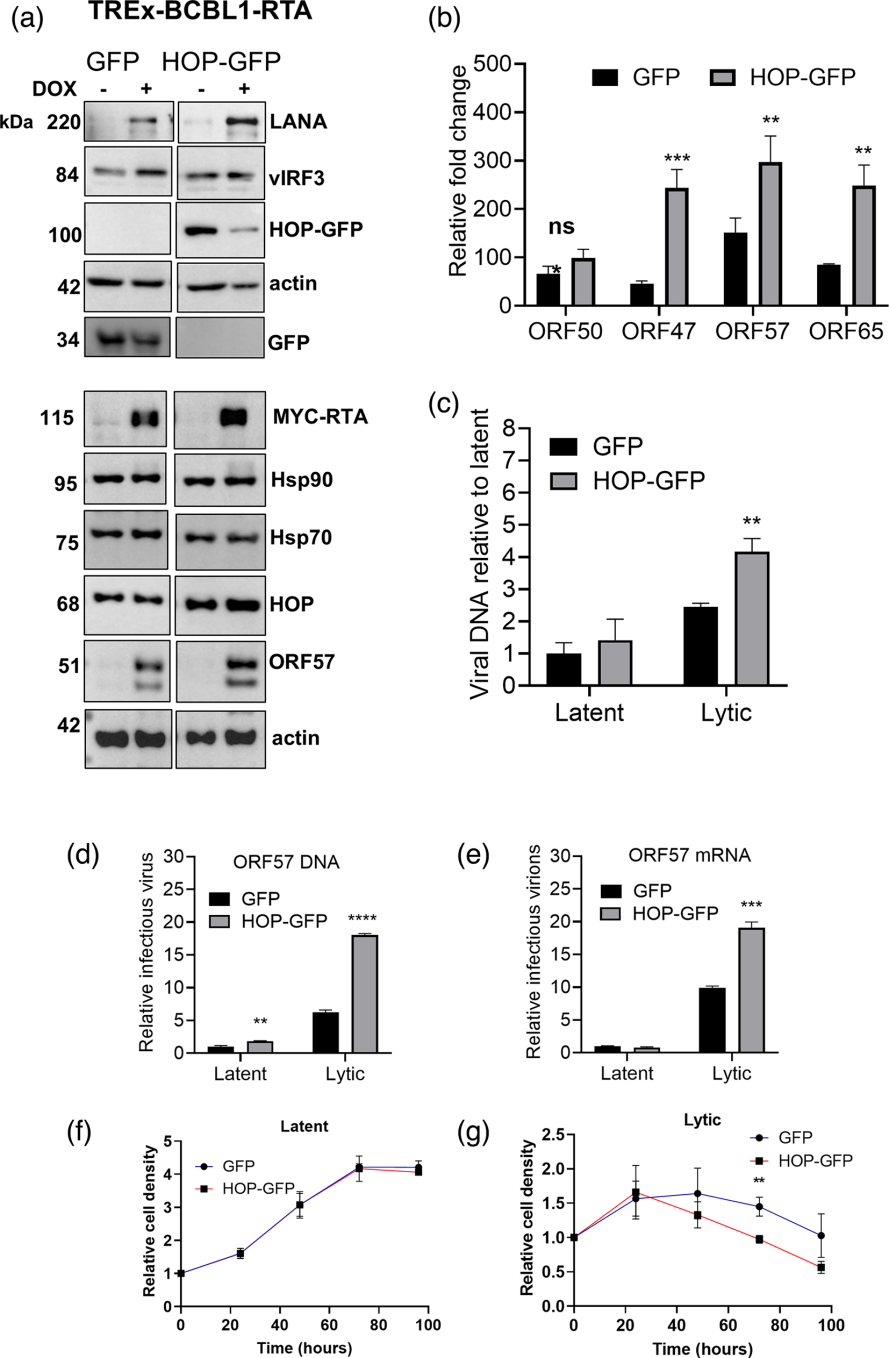
HOP overexpression increased KSHV lytic replication. (**a)** TREx-BCBL-1-RTA cell lines expressing GFP (control) or HOP–GFP were treated with doxycycline (dox) to induce lytic reactivation for 24 h before protein levels were confirmed by Western blot analysis. (b) Relative expression of viral genes in GFP and HOP–GFP cell lines at 24-h lytic reactivation. (c) Quantitation of viral load. TREx-BCBL-1-RTA cell lines were treated with dox to induce lytic reactivation for 72 h or left untreated (latent) and total DNA was isolated. Viral DNA load was assessed relative to host DNA by qPCR analysis. (d) Viral reinfection assay to quantify infectious virion production. TREx-BCBL-1-RTA cells were treated with dox (lytic) for 72 h to reactivate KSHV lytic replication or left untreated (latent). HEK293T cells were incubated with the culture medium from the untreated and dox-treated cells. Either viral DNA or RNA was measured by qPCR analysis compared to either host DNA or mRNA reference transcript. Proliferation of HOP–GFP or GFP expressing cell lines under (e) latent or (f) lytic reactivation. Data are averages (±sem, *n*=3). Unpaired t-tests were used to determine the statistical significance relative to GFP samples, where *, ** and *** indicate *P* values of <0.05, <0.01 and <0.001, respectively.

To confirm the effects of HOP on KSHV lytic replication, we next created the TREx-BCBL1-RTA cell lines expressing GFP (control) or GFP–HOP (Fig. 4a) and monitored KSHV lytic replication by assessing the levels of KSHV proteins (Fig. 4a), lytic transcript abundance relative to GAPDH by qPCR (Fig. 4b), viral DNA load by qPCR (Fig. 4c) and infectious virion production by reinfection assay (Fig. 4d). Consistent with our observations when HOP was depleted, the overexpression of HOP–GFP increased KSHV lytic replication. The reactivation of lytic replication increased LANA levels in the GFP control cell line, while the overexpression of HOP–GFP further increased LANA levels above the GFP control (Fig. 4a). HOP–GFP expression did not alter the levels of Hsp70 and Hsp90 chaperones but did lead to a minor increase in ORF57 protein levels compared to the GFP control (Fig. 4a). The expressions of viral lytic genes ORF47, ORF57 and ORF65 were significantly increased in HOP–GFP overexpressing TREx-BCBL1-RTA cells compared to those expressing GFP, despite equivalent levels of ORF50/RTA gene expression (Fig. 4b) and similar levels of inducible MYC–RTA (Fig. 4a). Interestingly, the significant increases in viral transcripts occurred despite a dramatic decrease in GFP–HOP protein levels during lytic replication compared to latency (Fig. 4a). This decrease may occur due to the silencing of HOP–GFP transcripts, which are expressed from an exogenous cytomegalovirus promoter compared to the endogenous expression of HOP, which was increased in WT TREx-BCBL-1-RTA cells at 48 h post-lytic reactivation (Fig. 1c). Nevertheless, the presence of overexpressed GFP–HOP at latency prior to the reactivation of lytic replication appeared sufficient to promote lytic replication.

HOP–GFP overexpression also significantly increased both viral DNA loads (Fig. 4c) and the production of infectious virions (Fig. 4d, e). There was only a 1.5-fold difference between latent and lytic stages for shNT TREx-BCBL1-RTA cells (Fig. 3d) and a sixfold difference for GFP TREx-BCBL 1-RTA cells (Fig. 4e), but the changes between conditions (shNT and shHOP, or GFP and HOP–GFP) were still evident. HOP–GFP overexpression consistently had a more potent effect on viral lytic replication than HOP depletion with greater fold changes observed (compare Figs3bd and 4b, c, e). HOP–GFP overexpression had no significant effect on cell growth during latent conditions compared to the GFP control but led to decreased cell growth during lytic replication from 72 h post-lytic reactivation. Taken together with Fig. 3c, d, this suggests that the presence of increased HOP could support viral lytic replication to consequently inhibit host cell growth.

Here, we provide the first data to show that HOP abundance influences the extent of KSHV lytic replication. HOP has been linked to the replication of the plant viruses potato virus Y (PYV) [57] and carnation Italian ringspot tombusvirus (CIRV). In contrast to KSHV, HOP depletion increased CIRV replication at the mitochondrial membrane [59] and HOP colocalized to ER-derived PYV replication complexes [57]. The data from these plant viruses suggest that HOP may act as either a pro- or antiviral host factor depending on context. Plants have three different HOP genes that produce distinct proteins, which retain the features required to interact with Hsp70 and Hsp90 but show distinct functions [60,61]. Additionally, PYV and CIRV are both RNA viruses and neither replicate in the nucleus like KSHV. Therefore, it is possible that the difference in the subcellular localization of the RNA RTC influences HOP’s function. Indeed, HOP could not block the replication of the tomato bushy stunt virus or cucumber necrosis virus, which are related to CIRV but replicate in peroxisome-derived membranes [59].

Cellular proteostasis must be sustained to support cell viability. Hsp90 and Hsp70 chaperone function regulates oncogenesis through the stabilization of oncoproteins, kinases and transcription factors important for malignancy [16] and is modulated by HOP [62]. Therefore, HOP may regulate KSHV replication indirectly via modulation of molecular chaperone complexes. Viral proteins must co-opt host chaperone systems to support viral proteostasis, which includes protein folding, translocation and degradation. Additionally, chaperones are important in functioning as buffers for mutations that arise in viral proteins. In this way, chaperone systems also support viral fitness and evolution [63]. While it is not known what proportion of viral proteins requires host chaperones, the largest classes of viral Hsp90 clients are viral DNA polymerases [10,49, 64]. Hsp90 is involved in the assembly of the KSHV replication complex [65]. Therefore, HOP may regulate viral replication by modulating Hsp90 complexes [49,64, 65]. This is supported by the fact that a peptide-based inhibitor of the HOP–Hsp90 interaction also blocks KSHV lytic replication [66]. Examples from viral host protein mimics demonstrate that viral proteins are often more sensitive to perturbations in proteostasis than host proteins [67]. Therefore, the potential for selective inhibition of viral protein homeostasis while retaining sufficient proteostatic capacity for host cell survival is a promising therapeutic opportunity for viral-driven cancers. However, issues with the clinical application of Hsp90 and Hsp70 inhibitors in cancer have increased interest in alternative strategies to target proteostasis complexes, one of which is targeting co-chaperones [68]. In KSHV infection, N-terminal pan-Hsp90 inhibitors have antiviral effects [13,15], but these also result in the stimulation of alternate stress pathways to upregulate Hsp70 and circumvent the effects of Hsp90 inhibition [4,13]. Therefore, the targeting of co-chaperones like HOP may present an effective option for the suppression of both the KSHV life cycle and viral-induced host oncogenic processes [31]. Numerous studies have suggested that HOP is a promising molecular target for cancer therapies because of its upregulation and ability to regulate multiple pro-oncogenic chaperones [69,71]. Although HOP is upregulated in viral-associated cancers like hepatocellular carcinoma where it has prognostic value [72], reports on the function of HOP in viruses and in viral-associated processes that drive these cancers are limited. HOP is essential for development in the mouse [73], but it can be knocked out of adult human cell lines without impacting cell viability [25]. A peptide targeting the HOP–Hsp90 interaction was able to block KSHV lytic replication without significantly reducing cell viability, showing proof of concept that HOP may be a druggable target [66]. This suggests that HOP null cell lines or cells treated with HOP inhibitors have sufficient proteostasis capacity to support cell survival but not viral replication.

## Conclusion

Taken together, our data demonstrate that HOP is a new host factor required for lytic replication of KSHV. HOP colocalized with LANA and the viral episome during lytic replication and changes in HOP abundance impacted the efficiency of lytic replication, without significantly impacting cell viability. HOP functioned at least in part by modulating the expression of KSHV lytic genes. However, the detailed mechanism by which HOP regulates KSHV biology is currently unknown and future experiments to describe this are ongoing. Since the KSHV lytic phase is important in viral oncogenesis and the development of KS, HOP could represent a novel host target for KSHV-related cancers in the future.

## Supplementary material

10.1099/jgv.0.002053 Uncited Table S1.

## Abbreviations

CIRV: carnation Italian ringspot tombusvirus
DMEM: Dulbecco’s Modified Eagle’s Medium
ER: endoplasmic reticulum
HOP: Hsp90–Hsp70-organizing protein
HSP: heat shock protein
KS: Kaposi’s sarcoma
KSHV: Kaposi’s sarcoma-associated herpesvirus
LANA: latency-associated nuclear antigen
LANA-NBs: LANA-associated nuclear bodies
PYV: potato virus Y
qPCR: quantitative polymerase chain reaction
RPMI: Roswell Park Memorial Institute
RTA: replication and transactivator
RTCs: replication and transcription centres
TBST: tris buffered saline with Tween 20
TPA: tetradecanoylphorbol acetate
