# Supplementary material for: Hsp70-Hsp90 organising protein (HOP/STIP1) is required for KSHV lytic replication
Source: J Gen Virol. 2024 Nov 28;105(11):002053. doi: 10.1099/jgv.0.002053 (PMC12453414; doi:10.1099/jgv.0.002053)
Supplement: Uncited Table S1. [file jgv-105-02053-s001.pdf]

HOP/STIP1 is required for KSHV lytic replication.

Elisa Kirigin[1], Michael Obinna Okpara[1], Lorraine Matandirotya[1,2], Jamie-Lee Ruck[1], Frederick Weaver[2], Zoe Jackson[2], Abir Chakraborty[1], Clinton Gareth Lancaster Veale[3], Adrian Whitehouse[1,2], Adrienne Lesley Edkins[1,4].

[1]Biomedical Biotechnology Research Unit (BioBRU), Department of Biochemistry and Microbiology, Rhodes University, Makhanda, 6139, South Africa
[2]School of Molecular and Cellular Biology, Faculty of Biological Sciences, University of Leeds, Leeds, LS2 9JT, UK
[3] Department of Chemistry, University of Cape Town, Rondebosch, Cape Town, 7700, South Africa
[4]Centre for Chemico- and Biomedicinal Research (CCBR), Rhodes University, Makhanda, 6139, South Africa

Corresponding author: Adrienne Edkins a.edkins@ru.ac.za

Supplementary Data

Table S1: Primer sequences used for qPCR analysis.

Target gene	Primer name	Direction	Sequence (5'-3')
GAPDH	GAPDH Hs.PT.39a.22214836	F	ACATCGCTCAGACACCATG
		R	TGTAGTTGAGGTCAATGAAGGG
Hop	Hop Hs.PT.58.22484332	F	AGCAGATCATGAGTGACCCA
		R	AATCAGACCCACCTCCATCAG
ORF57	pKSORF57q-F	F	CACTTCTGGAATACTACAGGCCAGG
	pKSORF57q-R	R	GTTAAATTTGGCCGACCCCATTGC
ORF73	pKSORF73q-F	F	CCAGACCAGTCGCCCATAACTTATTG
	pKSORF73q-R	R	GGAAGATTGTAGGTTTCCTGCCAGG
vIRF3	pKSvIRF3q-F	F	AGCCGTACACTGTGTTGATAC
	pKSvIRF3q-R	R	CACGATTCATAGTGAGGAACA
ORF45	pKSORF45q-F	F	CCGCCCACTCGATTTCATCAGG
	pKSORF45q-R	R	TCCAGCCACGGCCAGTTATATGC
ORF47	pKSORF47q-F	F	CGCGACCACTGCAGATAGCTCTATTC
	pKSORF47q-R	R	TTCCCTTTTGACCTGCGTGCG
ORF65	pAWKSORF65q-F	F	AAGGTGAGAGACCCCGTGAT
	pAWKSORF65q-R	R	TCCAGGGTATTCATGCGAGC
ORF50	pKSORF50q-F	F	AGACCCGGCGTTTATTAGTACGT
	pKSORF50q-R	R	CAGTAATCACGGCCCCTTGA

Table S2: Primary and secondary antibodies

Antibody	Host	Supplier	Catalogue No	Dilution
GFP (IgG1kappa) (clones 7.1 and 13.1)	Mouse	Sigma-Aldrich	11814460001	WB 1: 2500
Hop/STIP1	Rabbit	Abcam	ab126724	WB 1: 10000<br>IFA 1:250
Hsp90 α/β	Mouse	Santa Cruz	sc-13119	WB 1:5000
Hsp70/Hsc70	Mouse	Santa Cruz	sc-59571	WB 1:5000

β-actin	Rabbit	Abcam	ab213262	WB 1:2500
ORF57	Mouse	Santa Cruz	sc-135746	WB 1:5000
ORF73/LANA1 (4C11)	Mouse	Novus	NBP1-30176	WB 1:5000<br>IFA 1: 250
vIRF3/LANA2 (CM-A807)	Mouse	Novus	NB200-167	WB 1:2500
Anti-Myc tag antibody (Myc.A7)	Mouse	Abcam	ab94936	WB 1:2000
GAPDH HRP conjugated		Santa Cruz	Sc47724 HRP	WB 1:10000
Secondary anti mouse IgG H&L (HRP conjugated)	Goat	Abcam	ab150106	WB 1:1000
Secondary anti rabbit IgG H&L (HRP conjugated)	Donkey	Abcam	ab97064	WB 1:10000
Secondary anti-mouse Alexa Fluor 555	Donkey	Abcam	ab150106	IFA 1:500
Secondary anti-rabbit Alexa Fluor 488	Donkey	Invitrogen	A31573	IFA 1:500

Plasmids

The lentiviral packaging plasmid, psPAX2 (Addgene #12260- Didier Trono Lab) and the VSV-G envelope expressing plasmid, pMD2.G (Addgene #12259- Didier Trono Lab) were used for lentivirus particle development. For silencing of Hop, the shRNA used was the lentiviral vector plasmid, pGIPZ-shHOP or pGIPZ-shNT (Addgene) containing either shRNA targeting human HOP or a non-targeting control sequence. The overexpression plasmids pLJM1-GFP and pLJM-Hop1b-GFP encode GFP and Hop-GFP amino acid sequences, respectively, in the lentiviral pLJM1-eGFP backbone (Addgene #19319).
